# Identification of Candidate Adherent-Invasive *E*. *coli* Signature Transcripts by Genomic/Transcriptomic Analysis

**DOI:** 10.1371/journal.pone.0130902

**Published:** 2015-06-30

**Authors:** Yuanhao Zhang, Leahana Rowehl, Julia M. Krumsiek, Erika P. Orner, Nurmohammad Shaikh, Phillip I. Tarr, Erica Sodergren, George M. Weinstock, Edgar C. Boedeker, Xuejian Xiong, John Parkinson, Daniel N. Frank, Ellen Li, Grace Gathungu

**Affiliations:** 1 Department of Applied Mathematics and Statistics, Stony Brook University, Stony Brook, New York, United States of America; 2 Department of Medicine, Stony Brook University, Stony Brook, New York, United States of America; 3 Department of Pediatrics, Stony Brook University, Stony Brook, New York, United States of America; 4 Department of Pediatrics, Washington University St. Louis, St. Louis, Missouri, United States of America; 5 Department of Molecular Microbiology, Washington University St. Louis, St. Louis, Missouri, United States of America; 6 The Genome Institute, Washington University St. Louis, St. Louis, Missouri, United States of America; 7 Department of Medicine, University of New Mexico, Albuquerque, New Mexico, United States of America; 8 Program in Molecular Structure and Function, The Hospital for Sick Children, Toronto, Canada; 9 Department of Biochemistry & Molecular and Medical Genetics, University of Toronto, Toronto, Canada; 10 Department of Medicine, University of Colorado, Denver, Colorado, United States of America; Indian Institute of Science, INDIA

## Abstract

Adherent-invasive *Escherichia coli* (AIEC) strains are detected more frequently within mucosal lesions of patients with Crohn’s disease (CD). The AIEC phenotype consists of adherence and invasion of intestinal epithelial cells and survival within macrophages of these bacteria *in vitro*. Our aim was to identify candidate transcripts that distinguish AIEC from non-invasive *E*. *coli* (NIEC) strains and might be useful for rapid and accurate identification of AIEC by culture-independent technology. We performed comparative RNA-Sequence (RNASeq) analysis using AIEC strain LF82 and NIEC strain HS during exponential and stationary growth. Differential expression analysis of coding sequences (CDS) homologous to both strains demonstrated 224 and 241 genes with increased and decreased expression, respectively, in LF82 relative to HS. Transition metal transport and siderophore metabolism related pathway genes were up-regulated, while glycogen metabolic and oxidation-reduction related pathway genes were down-regulated, in LF82. Chemotaxis related transcripts were up-regulated in LF82 during the exponential phase, but flagellum-dependent motility pathway genes were down-regulated in LF82 during the stationary phase. CDS that mapped only to the LF82 genome accounted for 747 genes. We applied an *in silico* subtractive genomics approach to identify CDS specific to AIEC by incorporating the genomes of 10 other previously phenotyped NIEC. From this analysis, 166 CDS mapped to the LF82 genome and lacked homology to any of the 11 human NIEC strains. We compared these CDS across 13 AIEC, but none were homologous in each. Four LF82 gene loci belonging to clustered regularly interspaced short palindromic repeats region (CRISPR)—CRISPR-associated (*Cas*) genes were identified in 4 to 6 AIEC and absent from all non-pathogenic bacteria. As previously reported, AIEC strains were enriched for *pdu* operon genes. One CDS, encoding an excisionase, was shared by 9 AIEC strains. Reverse transcription quantitative polymerase chain reaction assays for 6 genes were conducted on fecal and ileal RNA samples from 22 inflammatory bowel disease (IBD), and 32 patients without IBD (non-IBD). The expression of Cas loci was detected in a higher proportion of CD than non-IBD fecal and ileal RNA samples (p <0.05). These results support a comparative genomic/transcriptomic approach towards identifying candidate AIEC signature transcripts.

## Introduction

Crohn’s disease (CD) is a form of inflammatory bowel disease (IBD) that is characterized by skip lesions of transmural inflammation, and can occur at multiple sites in the digestive tract. Inflammation can be found anywhere in the gastrointestinal tract from the mouth to the anus, but in most (60–80%) CD patients, the distal small intestine is frequently involved [1, 2]. Factors implicated in the pathogenesis of IBD include host genetic predisposition, and continual activation of the mucosal immune system by luminal bacteria and their products [3, 4]. From 16S ribosomal RNA gene sequence data, several laboratories have demonstrated imbalances in the gut microbial composition of CD patients, particularly those with ileal involvement when compared to unaffected individuals [5–16]. A consistent feature is a reduction in the relative frequency of *Faecalibacterium prausnitzii* [8] and an increase in Proteobacteria, particularly *Escherichia coli* [5]. A greater relative abundance of *E*. *coli* has been associated with CD, and particularly in active disease compared to patients in remission [17]. Mucosa-associated *E*. *coli* in particular are more abundant in CD [18] and in several small studies were isolated from inflamed tissue that include areas with ulcers and granulomas [19, 20]. In addition *E*. *coli* from the neoterminal ileum in post-surgical CD patients are linked to early recurrence of the disease [2].

Adherent invasive *E*. *coli* (AIEC) are considered to be pathobionts [21–23] and are isolated from the intestinal mucosa in humans with a higher prevalence in CD patients than in healthy subjects [2, 24, 25]. The AIEC phenotype requires adherence and invasion of intestinal epithelial cells and survival and replication within macrophages [26, 27]. Only a few commensal *E*. *coli* have been tested for this phenotype [28]. Using these methods, AIEC strains are detected in 22–52% of ileal CD patients and in 6–18% of non-IBD subjects [2, 18, 29–31]. However, these studies differ with respect to the number of biopsies analyzed, the anatomical location of the biopsies, and disease activity.

The design of a culture independent assay is hindered by the fact that although AIEC usually belong to the B2 or D groups, they are phylogenetically heterogeneous [18, 32]. Jensen et al [33] reported a quantitative real-time PCR (RT-qPCR) to determine the proportion of *E*. *coli* LF82 in DNA from human intestinal biopsies using spiked samples, but has not reported the results of this assay using clinical samples. Furthermore the genomic target of this assay, the pMT1-like plasmid, is not conserved among AIEC. Dogan et al,[34] reported that genes encoding processes responsible for propanediol utilization (*pdu* operon) and iron acquisition (yersiniabactin, *chu* operon) are overrepresented in human and dog AIEC genomes and might represent AIEC virulence factors.

To gain insight into biological pathways that contribute to AIEC pathogenicity we conducted a comparative transcriptomic analysis of the reference AIEC strain LF82 and the non-invasive commensal strain HS, grown in pure cultures. Furthermore, the genomic sequences of 11 non-invasive *E*. *coli* strains, including MG1655 [35] and HS [36], and a panel of 13 AIEC strains [34, 37–41] were compared to identify coding regions that could potentially serve as AIEC probes. Five of these gene targets and the previously described gene *pduC*, were tested by reverse transcriptase quantitative polymerase chain reaction (RT-qPCR) following extraction of RNA from fecal and ileal biopsy samples from 53 patients with and without IBD.

## Materials and Methods

### Homology searches in AIEC and non-invasive *E*. *coli* genomic sequences

The characteristics of seven previously published human AIEC (strains LF82, UM146, NRG857c, HM605, 541_1, 541_15, 576_1) and three human NIEC (strains T75, HS and MG-1655), are summarized in Table 1. Reference genomes were retrieved from NCBI [28, 34–41]. The characteristics of the six AIEC (strains MS-107-1, MS-115-1, MS-119-1, MS124-1, MS145-7, MS57-2), and 8 NIEC (strains MS185-1, MS187-1, MS196-1, MS198-1, MS45-1, MS60-1, MS78-1, MS84-1) are also listed in Table 1. These MS strains were isolated from de-identified surgical resection specimens collected at Mount Sinai School of Medicine [6] from CD, UC and non-IBD patients and characterized with respect to AIEC phenotype. The genomes of these 14 *E*. *coli* strains are accessible through the Human Microbiome Project database [42]. Homologous CDS were compared for these 13 AIEC and 11 NIEC. A search was also conducted among diarrheagenic (DEC) and extraintestinal (ExPEC) pathogenic *E*. *coli* (S1 Table) using the alignment tool BLASTN (version 2.2.28+). Homologous genes were defined as those with ≥85% sequence identity over 90 to 110% of the length of the query as previously described [37].

**Table 1:** Characteristics of 13 human AIEC and 10 non-invasive E. coli isolates. The AIEC phenotype was assessed using gentamycin protection assays of epithelial invasiona and survival within macrophages. The IBD affectation status was described as CD, UC or non-IBD. The anatomic site or source was the ileum, colon or feces. The pathology if available was described as macroscopically unaffectred or diseased or was not documented (-). For feces, the pathology was not applicable (N.A.) The K12-MG1655 strain was cured from K12 and has been maintained as a laboratory strain. The orginal K12 strain was isolated from a patient suffering from diphtheria.

E. coli strain	AIEC phenotype	IBD affectation status	Anatomic site	Pathology	Reference
LF82	AIEC	CD	ileum	diseased	[37]
NRG857c	AIEC	CD	ileum	-	[38]
UM146	AIEC	CD	ileum	-	[40]
HM605	AIEC	CD	colon	-	[39]
541_1	AIEC	CD	ileum		[34]
541_15	AIEC	CD	ileum		[34]
576_1	AIEC	CD	ileum		[34]
MS-107-1	AIEC	CD	ileum		
MS-115-1	AIEC	UC	colon	diseased	
MS-119-7	AIEC	CD	colon	-	
MS-124-1	AIEC	CD	ileum	unaffected	
MS-145-7	AIEC	CD	colon	-	
MS-57-2	AIEC	Non-IBD	ileum	unaffected	
HS	Non-invasive	Non-IBD	feces	N.A.	[36]
K12-MG1655	Non-invasive	Non-IBD	feces	N.A.	
T75	Non-invasive	CD	ileum	-	[34]
MS-185-1	Non-invasive	Non-IBD	colon	unaffected	
MS-187-1	Non-invasive	Non-IBD	colon	unaffected	
MS-196-1	Non-invasive	Non-IBD	colon	unaffected	
MS-198-1	Non-invasive	Non-IBD	colon	unaffected	
MS-45-1	Non-invasive	UC	colon	diseased	
MS-60-1	Non-invasive	Non-IBD	colon	diseased	
MS-78-1	Non-invasive	UC	colon	diseased	
MS-84-1	Non-invasive	CD	ileum	unaffected	

### Bacterial RNA isolation, sequencing and alignment to genomes

The reference AIEC strain LF82, originally isolated by Dr. Darfeuille-Michaud, was provided as a gift by Dr. Phillip Sherman (University of Toronto) and its identity was confirmed by multi-locus sequence typing[43]. The non-invasive HS strain was purchased from American Type Culture Collection (ATCC 700891). Triplicate Luria broth cultures (37°C) of LF82 and HS were grown with continuous shaking for 2 hours (exponential phase) and 24 h without shaking (stationary phase). Total RNA was extracted from the cells using the RiboPure Bacteria kit (Life Technologies Corp. Carlsbad, CA), following the manufacturer’s protocol. The average RNA Integrity Number (RIN) over all samples was 7. Two micrograms of RNA was depleted of ribosomal RNA using the RiboMinus Transcriptome Isolation Kit (Life Technologies Corp. Carlsbad, CA). These samples were then used as a template for strand-specific cDNA synthesis and subjected to single-end 150 bp Illumina sequencing. The RNA-Seq libraries were prepared and sequenced at the New York Genome Center (NYGC). Raw sequences were filtered to remove human sequence contamination, remove short reads (< 50 bp), depleted of duplicate reads, and quality trimmed using Trimmomatic (v 0.32) [44]. rRNA sequences were identified and culled using SortMe RNA (v1.9) [45]. Raw sequence reads for LF82 and HS were mapped to NCBI reference genomes NC_011993 and NC_009800, respectively [37] using the Burroughs Wheeler aligner (BWA) [46]. Counts for each annotated genomic loci were determined by HTseq-count (version 0.6.1) [47]. The data discussed in this publication have been deposited in NCBI's Gene Expression Omnibus and are accessible through GEO Series accession number GSE69020.

### Differentially Expressed Genes (DEGs) in LF82 compared to HS

Two DEG algorithms were employed, edgeR [48] and DESeq [49]. The raw counts produced by HTseq-count provided the input variables for the DESeq and edgeR packages. DEGs were defined as ≥ 2 fold change and FDR < 0.05 and LF82 and HS transcripts were compared at 2h or 24h, independently. DEGs resulting from edgeR were the input variables for knowledge based biological functions using the Gene Ontology (GO) plugin BiNGO [50] and the custom ontology and annotation files found on the Gene Ontology website [51, 52]. DEGs resulting from DESeq were the input variables for knowledge based pathways/modules defined either by the Kyoto Encyclopedia of Genes and Genomes (KEGG, http://www.genome.jp/kegg/) [53] or a set of modules obtained through clustering a network of high quality functional interactions predicted for *E*. *coli* [54]. The up-regulated and down-regulated output from DESeq for each time point were entered to identify the perturbed pathways regardless of the overall polarity.

### Ethics Statement

This study was approved by the Institutional Review Board (IRB) at Stony Brook University Hospital. Pediatric (age ≥ 7 years) and adult patients are recruited in a consecutive fashion by the Stony Brook Digestive Diseases Research Tissue Procurement Facility and provide verbal and written consent for chart abstraction, blood, stool, tissue biopsies and/or surgical waste collection with analysis for research purposes and for their information to be stored in the hospital database. For children between 7–17 years old participating in this study, both oral and written parent/legal guardian permission and a separate oral and written assent from the child was obtained. The IRB at Stony Brook University Hospital approved this consent procedure.

### Enrollment of patients and collection of samples

After receiving IRB approval, participants previously scheduled to undergo colonoscopy or intestinal resection, were identified and consented. Pediatric (ages ≥7 years) and adult patients were recruited in a consecutive fashion by the Stony Brook Digestive Diseases Research Tissue Procurement Facility. The period of enrollment was between March 2011 and June 2014. Patients with a confirmed diagnosis of IBD were phenotyped based on endoscopic and radiographic studies as previously described [55]. Tissue specimens were collected and immediately placed into RNAlater (Life Technologies, Carlsbad, CA).

### DNA isolation from bacteria

Nine bacterial strains were processed for DNA isolation: LF82, MG1655, HS, and 6 MS AIEC strains. Following overnight culture, a single colony of each bacterial strain was placed in 5 ml of tryptic soy broth and incubated overnight at 37°C with shaking. Total bacterial DNA was extracted using the QIAamp DNA Mini Kit and according to the manufacturer’s protocol and stored at -20°C until batch analysis.

### PCR and electrophoresis

The forward and reverse primers for the *Cas* genes (strains LF82_088, LF82_091, LF82_092 and LF82_093) were designed using the NCBI primer designing tool Primer-BLAST [56]. The *E*. *coli* 16S rRNA forward and reverse primers were previously validated [57]. The predicted PCR products were 340 bp for *E*. *coli* 16S rRNA, 107 bp for LF82_088, 109 bp for LF82_091, 97 bp for LF82_092, and 125 bp for LF82_093. Amplification was performed in a 15 μL reaction volume and consisting of 1.5 μL 10X PCR buffer (Qiagen), 3 μL Q solution, nuclease free water, 0.5 μM forward and reverse primers, 0.1 uL Qiagen Taq DNA polymerase, and 1μL template. PCR was performed using an Eppendorf Mastercycler EPGradient S. The following thermal cycling conditions were used: 5 min at 94°C and 36 cycles of amplification consisting of 30 seconds at 95°C, 30 seconds at 56°C, and 1 min at 72°C, with 5 min at 72°C for the final extension. PCR product bands were analyzed after electrophoresis in a 1% agarose gel in 1X TBE containing ethidium bromide and digital imaging using The ChemiDoc MP system (Biorad, Hercules, CA).

### RNA isolation from stool and bacteria

Total bacterial RNA was extracted from each stool sample using a fecal RNA isolation kit (Zymo Research Corporation, Irvine, CA) according to the manufacturer’s protocol. RNA from strains LF82, MG1655, and HS was extracted using the same kit after culture for 2 and 24 hours. RNA was archived at -80^0^ C until batch analysis.

### RNA isolation from ileal biopsies

Fresh frozen ileal biopsies were homogenized individually in 2 ml of Trizol solution (Life Technologies) with the PowerGen125 homogenizer (Fisher Scientific) and 1 ml aliquots placed into 1.5 mL microcentrifuge tubes. RNA was subsequently extracted using phenol/chloroform extraction methods as previously described[58]. The RNA was reconstituted in 50ul of RNA Storing Solution (Life Technologies) and stored at -80^0^ C until batch analysis.

### Reverse transcription quantitative polymerase chain reaction (RT-qPCR) of *E*. *coli* transcripts

For cDNA production, 500 nanograms of RNA was added to a 20 μL reaction using the SuperScript VILO cDNA Synthesis Kit (Life Technologies, Carlsbad, CA). Quantitative PCR was conducted in triplicate on 1:2 dilutions of cDNA from fecal samples and 1:2, 1:4 and 1:8 dilutions of cDNA from pure *E*. *coli* cultures and using 1 μL volumes. Amplification was performed in a 20 μL reaction volume and consisting of 10 μl of 2x SYBR Green Master Mix, 1 μl each of 10uM forward and reverse primers, 1 μL of cDNA, and 7 μL of nuclease free water. The thermal cycling conditions were: 10 min at 95°C and 40 cycles of amplification consisting of 30 seconds at 95°C and 60 seconds at 60°C using a Mastercycler EPGradient S (Eppendorf). Primers included Total bacteria and *E*. *coli* 16S rRNA forward and reverse primers as previously validated [57] and the *pduC* gene as previously described [34]. Primers were designed for 5 candidate genes LF82_088, LF82_091, LF82_092, LF82_093, and LF82_095, using an online primer design tool[56]. The sequences of all primers are listed in S2 Table.

### Statistical analysis

All analyses were performed using the GraphPad Prism 5 software suite (GraphPad, San Diego, CA). For each RT-qPCR assay, the average cycle threshold (Ct) of 3 replicates per gene was determined. Positive assays had a mean threshold cycle values (Ct) ≤35. The Ct values in negative samples and water ranged from 39–40. Fisher’s exact test was performed to compare positive and negative counts in IBD compared to non-IBD and CD compared to non-IBD, for fecal and ileal biopsy samples, respectively. The relative abundance of *E*. *coli* 16S rRNA transcripts was determined by defining the delta Ct (ΔCt). ΔCt was generated by subtracting the average Ct value for total bacteria away from the average Ct value of *E*. *coli* 16S rDNA. The nonparametric Mann-Whitney test was used to compare values for IBD compared to non-IBD and CD compared to non-IBD for fecal and ileal biopsy samples, respectively.

## Results

### Identification of differentially expressed genes (DEG) in LF82 vs. HS

We analyzed gene expression levels of strains LF82 and HS in separate samples prepared from exponential (2h) and stationary (24h) phase cultures grown at 37°C, in order to interrogate gene expression under different growth conditions. Expression levels were standardized by reads per kilobase of exon per million mapped sequence reads (RPKM) [59]. The edgeR and the DESeq algorithms yielded similar findings. Results generated using edgeR are shown in S1 File. For the 2h and 24h samples, 654 and 459 CDS, respectively, had increased expression (RPKM ≥2 fold, FDR <0.05) in LF82 compared to HS (Table A in S1 File), with 224 of the CDS exhibiting increased expression in LF82 at both time points. At 2 h, 6 genes shared by LF82 and HS were expressed only in LF82. Similarly at 24 h, 17 genes had detectable transcripts in LF82 and not in HS (Table A in S1 File). Six genes were detected only in LF82 at both time points. Some of these genes are involved in bacteriophage infections and others have no known function (Table 2). A total of 712 and 492 genes had decreased expression at 2h and 24h respectively, in LF82 compared to HS (Table B in S1 File), with 241 genes exhibiting decreased expression in LF82 (RPKM ≤ 0.05, FDR <0.05) at both time points.

**Table 2:** These 6 CDS are homologous in LF82 and HS but transcripts are detected only in LF82 at both 2h and 24 h. The LF82 NCBI Locus Tags and the bacterial gene names (if available) are shown. The mean normalized RPKM at 2h and 24 h is shown.

LF82 NCBI Locus Tag	Gene	Function	Bacteria with identical protein	RPK 2h	RPK 24h
LF82_119		phage NinH protein	*Escherichia*	92.9	19.6
LF82_121		Holin–pore forming protein	*E*. *coli*, *Salmonella enterica* subsp. Enterica, *S*. *flexneri* bacteriophage	62.8	6.8
LF82_126		hypothetical protein	*E*. *coli*, *Shigella*	111.6	13.2
LF82_134		Phage head assembly protein	*E*. *coli*, *Salmonella*, *S*. *flexneri*	47.1	6.6
LF82_135		DNA transfer protein	*E*. *coli*, *Salmonella*, *Shigella*, *Cronobacter* bacteriophage	109.7	7.0
LF82_2871	*ydiE*	inorganic ion transport and metabolism	*E*. *coli*	44.2	47.4

**Fig 1:**
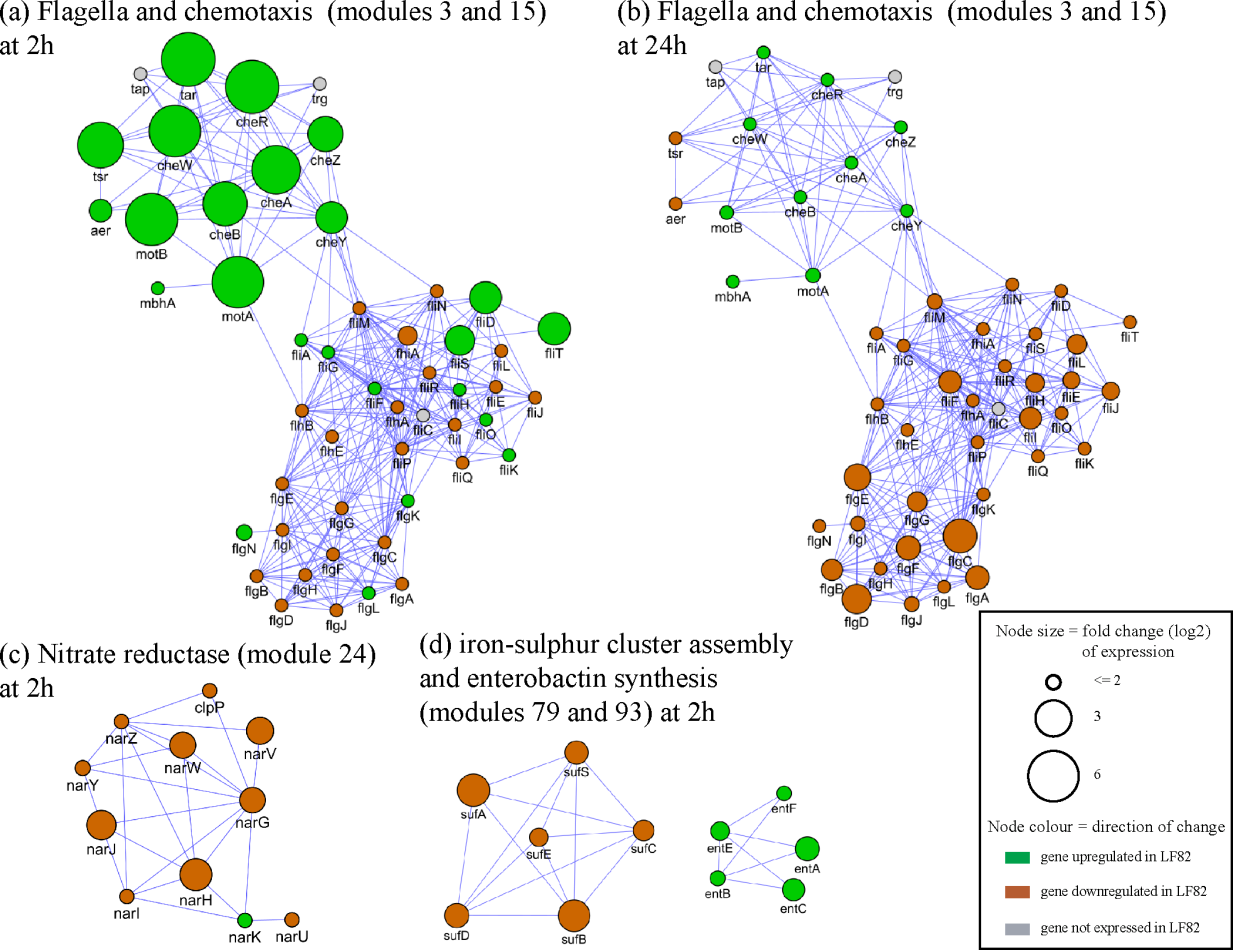
Functional modules differentially expressed in strain LF82 compared to non-invasive strain HS at 2h and 24h time points. As mentioned above, the differences can not be related to the duration of culture vs. the agitation. Functional modules were obtained from a high quality set of protein-protein interaction previously defined for *E*. *coli* [36]. Node size indicates fold change (log2) of differential expression (DE) based on the DESeq algorithm [33]. Node color indicates direction of regulation. Flagella and chemotaxis module: (a) most chemotaxis genes are up-regulated in LF82 relative to HS at 2h (DE > 3), while at 24h (b), half of the flagella genes are down-regulated in LF82 (DE > 2). (c) Most nitrate reductase genes are down-regulated in LF82 at 2h (DE > 2). (d) At 2 hours, iron-sulphur cluster assembly genes in LF82 are down-regulated (DE > = 2.7), and enterobactin synthesis genes (involved in iron transport) are up-regulated (DE > = 2.3).

Functional profiling of genes was accomplished using the Gene Ontology (GO) plugin BiNGO [50] and the custom ontology and annotation files on the Gene Ontology website (http://www.geneontology.org). This analysis revealed that multiple functional categories have overlapping datasets as shown in Tables A-D in S2 File. Examples include “siderophore metabolic process like enterobactin”, which are up-regulated at both time points, and”glycogen metabolic process” and “oxidation-reduction process”, which are down regulated at both time points (Table 3). Analysis using alternative pathways/modules gene sets [53, 54] facilitated visualization of patterns of gene expression against a very complex background. For example, the functional category chemotaxis is up-regulated (FDR = 0.008) in LF82 at 2h, but bacterial-type flagellum-dependent cell motility is down regulated (FDR = 1.4 x 10^−6^) at 24h. However as shown in Fig 1, the polarity of the DEGs are preserved at both time points. These network-based results draw attention to modules that do not overlap with the GO categories (e.g. modules 24 and 79 in Fig 1).

**Table 3:** Selected common up-regulated and down-regulated biological pathways in LF82 at 2h and 24h time points. For more comprehensive lists of up-regulated and down-regulated pathways at 2h and 24h please see **S2A and S2B Table**. The false discovery rate (FDR) is indicated for both the 2h and 24h cultures.

GO–ID	Pathway Genes at 24h time point	2h FDR	24h FDR
	***Up-regulated pathways***		
41	Transition metal transpor*t*	0.0017	0.0045
	*COPA|FIU|FEPA|FEOB|FES|FEPE|RCNA|YCDO|ZNUA|YCDB|MODC|CUSC|YDAN|CUSB*		
9247	Siderophore metabolic process	0.00035	0.0020
	*ENTE|ENTF|ENTC|YBDZ|ENTA|FES|ENTB*		
	***Down-regulated pathways***		
5977	Glycogen metabolic process	0.0031	0.043
	*GLGS|GLGC|GLGA|GLGX|GLGP*		
55114	Oxidation-reduction process	0.0000022	0.00018
	*TDCE|HYCF|MAEB|YEIA|TDCD|HYCD|HYCE|FUMA|FUMC|FUMB|YGHZ|YEIT|ASPA|NRFC|METF|TPX|YPHC|YDJL|NARH|PDXB|PFLA|YDBK|GLTA|YOAE|ARGC|GLGC|HYBC|MELA|HYBA|HYBB|HYBO|FADB|FADA|FADE|GLGP|NUOK|GLGS|SUCB|SUCA|SUCD|SUCC|YAJO|FDOI|TRXA|GLGX|FDOG|TRXC|FDOH|YGCO|UBID|UBIF|YHBW|YGCN|XDHA|NAPH|GND|FRE|NAPF|XDHB|XDHD|DADA|NAPA|YBDH|YJJN|RSPB|YDHU|ILVC|NDH|YDHV|GLGA|TYRA|YGGP|STHA|FADJ|ALLD|YHJA|LIPA|HDHA|IDND|FUCO|LPD|SDHA|YGFK|YBAE|DSBG|DUSA|SDHC|PUTA|YGFM|SDHD|YFEX|YDEP|UCPA|YDEM*		

### Selection of candidate AIEC signature transcripts

To identify candidate AIEC signature transcripts, we took a subtractive approach to identify coding DNA sequences that were present in the genome of the reference AIEC strain LF82 but not homologous to sequences in 11 non-invasive *E*. *coli* strains. In addition to the HS and MG1655 strains, we included 9 strains from patients with and without IBD and phenotyped with respect to their inability to invade epithelial cells and survive within macrophages. Although five of the non-invasive strains were isolated from non-IBD patients, four others were isolated from IBD patients (2 UC, 2 CD) (Table 1). Of the 4508 predicted CDS in the LF82 genome [37], 3446 could be uniquely mapped to corresponding CDS with ≥ 85% sequence identity in the control HS genome. Although 747 LF82 CDS lacked homology to the HS genome [36] further subtraction was accomplished by including 10 additional NIEC. In the final analysis, 166 CDS in LF82 were absent from all 11 NIEC genomes.

We compared the 166 CDS across six published AIEC genomes (UM146, NRG857c, HM605, 541–1, 541–15, 576–1) and six MS AIEC strains (MS-107-1, MS-115-1, MS-119-1, MS124-1, MS145-7, MS57-2). None of the 166 CDS were homologous to all 13 human AIEC genomes (see S1 Table). The CDS LF82_95, which encodes an excisionase, was the most prevalent with homology in 9 of 13 AIEC genomes (see Table 4 and S1 Table). This CDS also shared homology with a number of pathogenic *E*. *coli*, particularly DEC (see S1 Table). The CDS LF82_332 corresponds to the *pduC* gene and was homologous with 6 of 13 AIEC. We also selected 4 CDS (LF82_089, LF82_091, LF82_091, LF82_092, and LF82_093) that mapped to a region previously described as “specific region 6” [37] and corresponded to 4 CRISPR-Cas genes. Three AIEC (LF82, NRG857c, and MS-57-2) shared homologous CDS with all 6 candidate AIEC transcripts. Three AIEC strains 541_1, 576_1, and MS–115–1), shared only the *pduC* gene and 3 additional AIEC strains (UM146, HM605, and MS-145-7) shared only the 4 Cas genes.

**Table 4:** LF82 transcripts that share homology with at least 4 other AIEC genomes but none of the 11 NIEC genomes. (See also **S3 Table**). Putative protein function is based on sequence homology as listed in NCBI GENE. The mean RPKM are show in the 2h and 24 h LF82 cultures. The number of AIEC strains (total of 13) with CDS sharing >85% sequence homology is listed. The genes selected for exploratory RT-qPCR analysis of patient samples are in **bold.**

RefSeq ID	Putative protein	2h RPKM	24h RPKM	No. AIEC
**LF82_095**	**excisionase**	**10.3**	**8.4**	**9**
**LF82_088**	**CRISPR/Cas system-associated protein Cas1**	**11.3**	**28.3**	**6**
LF82_089	CRISPR/Cas system-associated protein Cas3/Cas2	22.2	39.9	6
**LF82_092**	**CRISPR/Cas system-associated RAMP superfamily protein Csy3**	**29.5**	**31.8**	**6**
**LF82_093**	**CRISPR/Cas system-associated RAMP superfamily protein Cas6f**	**12.0**	**9.7**	**6**
LF82_328	cobalamin biosynthesis protein CbiG	16.7	13.0	6
LF82_330	propanediol diffusion facilitator	0.7	3.3	6
LF82_331	propanediol utilization protein:polyhedral bodies	0.2	0.7	6
**LF82_332**	**propanediol utilization protein: glycerol dehydratase large subunit (*pduC*)**	**0.5**	**2.4**	**6**
LF82_333	propanediol utilization protein: diol dehydratase medium subunit	1.1	4.0	6
LF82_334	propanediol utilization protein: diol dehydratase small subunit	0.6	1.3	6
LF82_335	propanediol utilization protein: diol dehydratase reactivation	0.6	2.8	6
LF82_336	propanediol utilization protein: diol dehydratase reactivation	1.4	3.3	6
LF82_337	propanediol utilization protein: polyhedral bodies	9.1	13.6	6
LF82_338	propanediol utilization protein: polyhedral bodies	1.0	2.3	6
LF82_339	propanediol utilization protein	0.5	3.5	6
LF82_340	propanediol utilization protein	1.4	5.5	6
LF82_341	propanediol utilization protein: polyhedral bodies	1.1	2.9	6
LF82_342	propanediol utilization protein: B12 related	1.5	2.9	6
LF82_343	CoAdependent proprionaldehyde dehydrogenase	2.1	4.5	6
LF82_344	propanediol utilization protein: propanol dehydrogenase	0.6	0.9	6
LF82_345	propanediol utilization protein	1.0	2.9	6
LF82_346	propanediol utilization protein: polyhedral bodies	2.1	4.1	6
LF82_347	propanediol utilization protein: polyhedral bodies	2.2	3.5	6
LF82_778	putative propanediol utilization protein	0.2	3.4	6
LF82_013	hypothetical protein	12.8	12.5	5
LF82_090	hypothetical protein	17.7	13.2	5
LF82_199	iron compound ABC transporter	0.2	0.9	5
LF82_348	propanediol utilization protein	3.8	6.1	5
**LF82_091**	**CRISPR-associated protein (Cas_Csy2)**	**14.4**	**6.2**	**4**
LF82_329	Pdu/cob regulatory protein	7.3	10.8	4
LF82_389	variable tail fibre protein	0.7	3.2	4
LF82_441	hypothetical protein	9.1	21.9	4
LF82_548	major fimbrial subunit	11.3	21.3	4
LF82_550	outer membrane usher protein lpfC precursor	33.1	51.7	4
LF82_551	fimbrial chaperone protein	0.2	0.9	4
LF82_552	fimbrial-like protein	3.6	3.5	4
LF82_723	DHA kinase PgdK (EC 27129)	15.6	16.7	4
LF82_724	dihydroxyacetone kinase PdaK (EC271 29)	1.4	5.4	4
LF82_725	glycerol dehydrogenase CgrD (EC1116)	8.8	5.3	4
LF82_726	transporter CgxT	1.3	2.3	4
LF82_727	dihydrolipoamide dehydrogenase CdlD	3.5	5.4	4
LF82_728	carnitine transporter CniT	3.0	7.9	4
LF82_729	glycerate kinase GclK	2.4	6.6	4
LF82_730	3hydroxyisobutyrate dehydrogenase GhbD(EC 11131)	1.9	2.9	4
LF82_731	regulatory protein GclR	1.8	3.1	4
LF82_732	glycoxylate carboligase GclA	0.6	1.7	4
LF82_733	regulatory protein IbgR	4.7	6.0	4
LF82_734	Invasion protein IbeA	2.5	4.5	4
LF82_735	transporter IbgT	1.3	3.0	4

To test the *in silico* results and validate the PCR primers we amplified DNA for each of the 4 candidate genes (S1 Table). Agarose gel electrophoresis of PCR reactions verified amplification products of the expected sizes (see methods) for candidate genes LF82_091, LF82_092, LF82_093 and LF82_088 in strains LF82, MS145-7, and MS57–2 (S2 Table). All other strains, including MG1655, HS and the 4 MS AIEC strains without homologous Cas genes, exhibited no PCR amplification with these primers. All samples produced the expected band at 340 base pairs for the *E*. *coli* 16S rRNA gene product (S1 & S2 Figs).

### Screening Candidate Gene Transcripts in Human Clinical Specimens

RNA was isolated from fecal samples collected from 53 individuals at Stony Brook University. Within this collection, 43 (81.1%) stool samples were acquired from children (Table 3). Twenty-two were IBD patients and 31 individuals were non-IBD controls. Non-IBD patients included subjects with functional GI disorders, Celiac disease, lactose intolerance and one patient with juvenile polyps. The number of male patients was significantly higher in both IBD cohorts compared to controls, p = 0.009 and 0.029 for CD and UC respectively. CD patients were significantly older (p = 0.024). The median ages for CD, UC/IC and controls were 20, 16 and 15 years, respectively. The IBD patients included 14 patients with CD, 6 patients with UC and 2 with indeterminate colitis (IC). Three of the CD patients were diagnosed at enrollment. Parallel ileal biopsies were available for 10 CD patients, 3 UC patients and 23 non-IBD controls. Table 5 displays the characteristics of all subjects. For IBD patients, age of diagnosis, disease location and disease behavior (CD) are as defined by the Montreal classification[60]. Also included are disease duration, body mass index (BMI), smoking, surgical management of IBD, and IBD medications.

**Table 5:** Clinical characteristics of CD, UC/IC and non-IBD patients.

	CD N = 14	UC/IC N = 8	Non-IBD N = 32
Gender			
Male	11 (85%)	7 (88%)	11 (35%)
Age of Diagnosis, (Montreal A)			
A1 (≤16 yr)	71.4	87.5	
A2 (17–40 yr)	28.5	12.5	
A3 (>40 yr)			
Disease Location, CD (Montreal L)			
L1 ileal	21.4		
L2 colonic	7.1		
L3 ileocolonic	71.4		
Disease Location, UC (Montreal E)			
E1 proctitis		12.5	
E2 left-sided			
E3 extensive		83.0	
Disease Behavior, CD (Montreal B)			
B1 nonstricturing, nonpenetrating	57.1		
B2 stricturing)	21.4		
B3 penetrating—excludes perianal	21.4		
Median age at procedure (IQR)^a^ y	20 (14.2–25.7)	16 (12.7–17.2)	15 (11–17)
Median duration of disease (IQR) y	4.5 (1.4–6.8)	1.5 (0–10)	
Race			
Caucasian	11 (85%)	6 (75%)	28 (88%)
Current Smoker	1	0	1
Median BMI (IQR) kg/m2	21.0 (17–24)	19.8(18.7–22)	20.1(17.5–24.5)
Medications			
Mesalamine^b^	5 (36%)	1 (12%)	0
Steroids	2 (14%)	1 (12%)	
Immunomodulators^c^	4 (29%)	1 (12%)	
Anti TNF alpha biologics^d^	7 (50%)	1 (12%)	

^a^IQR: Interquartile range

^b^Mesalamine: Balsalazide, Mesalamine, Olsalazine, Sulfasalazine

^c^Immunomodulators: Imuran, Methotrexate

^d^Biologics: Adalimumab, Certolizumab, Infliximab

To compare the relative abundance of *E*. *coli* between clinical specimens, we performed RT-qPCR with *E*. *coli*-specific 16S rRNA gene primers and normalized results to total bacterial 16S rRNA gene expression (Tables 6 & 7). The median ΔCT values (Total-*E*. *coli* Ct) among CD, UC/IC and non-IBD fecal samples were -14.40, -7.14, and -13.56, respectively. There was no statistically significant difference in *E*. *coli* abundance compared to non-IBD controls. Among ileal biopsy specimens, the mean ΔCT values for CD, UC/IC and non-IBD samples were -9.94, -10.50, and -11.84, respectively. There was no statistically significant elevation in *E*. *coli* abundance in IBD specimens compared to controls.

**Table 6:** Fecal RT-qPCR results for candidate AIEC transcripts. The number of positive fecal stool samples are shown for each candidate AIEC transcript. Transcript is defined by LF82 locus tag and hypothetical function. Fisher’s exact tests were used to compare the frequencies of positive results. “*” represents P values of <0.05. The median ΔCt_E. coli_ (range) is shown. The nonparametric Mann-Whitney test was used to compare values for IBD compared to non-IBD and CD compared to non-IBD for fecal and ileal biopsy samples, respectively.

Transcript		CD	UC/IC	Non-IBD	P value	P-value
		N = 14	N = 8	N = 32	IBD vs. non-IBD	CD vs. Non-IBD
LF82_095	excisionase	9	4	14	0.41	0.34
LF82_332	*pduC*	3	3	8	1.00	1.00
LF82_088	*cas1_I-F*	5	1	2	0.05	0.02*
LF82_091	*cas_Csy2*	5	1	2	0.05	0.02*
LF82_092	*csy3_I-F*	5	1	2	0.05	0.02*
LF82_093	*cas6_I-F*	1	0	1	1.00	0.52
Median ΔCt_E. coli_	(IQR)	-14.4	-7.4	-13.6	0.48	0.81
		(-17.54 to -11.85)	(-12.81 to -4.80)	(-18.10 to -11.41)		

**Table 7:** Ileal RT qPCR results for candidate AIEC transcripts. The number of positive ileal biopsy samples are shown for each candidate AIEC transcript. Transcript is defined by LF82 locus tag and hypothetical function. Fisher’s exact tests were used to compare the frequencies of positive results. “*” represents P values of <0.05. The median ΔCt_E. coli_ (interquartile range) is shown. The nonparametric Mann-Whitney test was used to compare values for IBD compared to non-IBD and CD compared to non-IBD for fecal and ileal biopsy samples, respectively.

Transcript		CD	UC/IC	Non-IBD	P value	P-value
		N = 12	N = 3	N = 23	IBD vs. non-IBD	CD vs. non-IBD
LF82_095	excisionase	4	0	0	0.74	.0095*
LF82_332	*pduC*	2	0	1	0.55	0.27
LF82_088	*cas1_I-F*	9	2	11	0.09	0.16
LF82_091	*cas_Csy2*	6	0	3	0.12	0.04
LF82_092	*csy3_I-F*	6	0	3	0.12	0.04
LF82_093	*cas6_I-F*	3	0	0	0.05	0.03
Median ΔCt_E. coli_	(IQR)	-9.9	-10.5	-11.8	0.08	0.12
		(—11.92 to -7.35)	(-10.89 to -6.83)	(-16.56 to -9.60)		

The threshold of detection of transcripts corresponding to excisionase (LF82_095), *pduC* (LF82_332) and four Cas homologous genes (LF82_088, LF82_091, LF82_092, and LF82_093 was set at Ct ≤ 35. The negative Ct values ranged between 39 and 40. A higher proportion of CD fecal (Table 6) and ileal (Table 7) cDNA samples were positive for LF82_091 and LF82_092 transcripts than non-IBD fecal and ileal RNA samples (p <0.05) A higher proportion of CD fecal samples were positive for LF82_088 in CD vs. non-IBD samples and a higher proportion of CD ileal samples were positive for LF82_093 AND LF82_095 in CD vs. non-IBD.

The median ΔCT values (Total-*E*. *coli* Ct) among CD, UC/IC and non-IBD fecal samples were -14.40, -7.14, and -13.56, respectively. There was no statistically significant difference in *E*. *coli* abundance when compared to non-IBD controls. Among ileal biopsy specimens, the mean ΔCT values for CD, UC/IC and non-IBD samples were -9.94, -10.50, and -11.84, respectively. There was no statistically significant elevation in *E*. *coli* abundance in IBD specimens compared to controls.

## Discussion

Although a higher proportion of CD patients harbor AIEC, such organisms can also be recovered from non-IBD patients. Conversely, NIEC strains are recovered from IBD patients (Table 1). The pathogenic potential of AIEC may vary depending on host susceptibility. Host factors such as IBD risk alleles and Paneth cell function have been linked to alterations in ileal mucosa-associated microbial composition and the *Escherichia/Shigella* genus [12, 14, 57, 61, 62]. *In-vitro* analysis has not been performed for many human commensal *E*. *coli* strains. In this study the complete genomes for 13 AIEC and 11NIEC, all with prior *in-vitro* phenotypic analysis were compared.

Multiple studies have demonstrated that CD patients, particularly those with ileal disease, have altered intestinal microbial biodiversity and composition. Because most of these studies are based on 16S rRNA sequence analysis, they do not address alterations in microbial function, or in subgroups within identified species. Shotgun bacterial DNA metagenomics and bacterial metatranscriptomics measure alterations in microbial function more directly than does 16S rRNA sequence analysis. The advantage of bacterial transcriptomic data over shotgun metagenomics data is that the former provides information on which bacterial genes are actually transcribed. In this study we compared the transcriptomes of a reference AIEC strain, LF82 to a control strain HS to identify genes associated with the AIEC phenotype. We selected HS as the control strain which was previously demonstrated to be non-invasive [28].

A comparative analysis of genes shared between the LF82 and HS genomes indicated that many of the DEG had a relatively low fold change (~ 2–4 fold) making them less suited for clinical assays. Up-regulated genes in LF82 are involved in many key pathways including iron metabolism, supporting the recent report that AIEC strains are enriched for genes involved in iron utilization [37], a feature of many B2 phylotype members. Comparison of the transcriptional profiles revealed a significant effect of growth conditions (see Fig 1). We identified six genes with no detectable expression in HS (Table 2) at both growth conditions. Four of the genes code for identical proteins in the enteropathogenic bacteria *Salmonella* and *Shigella*. Further characterization of these proteins in AIEC and non-invasive *E*. *coli* strains is necessary to determine if they are a component of the AIEC phenotype.

In the comparative analysis of RNA-seq data, 747 CDS that mapped to the LF82 genome did not share homology with CDS in HS (S1 Table). We extended our comparative analysis to 13 *E*. *coli* strains with the AIEC pathotype and 11 NIEC (Table 1). Using a subtractive genomics approach, we found that the 166 CDS present only in LF82 were not homologous in all 11 NIEC (S1 Table). However, none of the 166 CDS were present in the 13 AIEC strains surveyed. This observation supports the concept that the AIEC pathovar is formed by a heterogeneous collection of serogroups and serotypes. As shown in Table 3, AIEC genomes are enriched in genes belonging to the *pdu* operon, the *ibe* operon, and the type VI secretion system [34, 37, 38]. The *pdu* operon is a component of a metabolic pathway required for fucose utilization [63], and is present in enterpathogenic bacteria and offers a competitive advantage for energy production under anaerobic conditions [34, 63]. The *ibeA* gene (invasion of brain endothelium) encodes an invasion protein found in several extraintestinal pathogenic *E*. *coli* (ExPEC) strains[64]. This gene may also play a role in *E*. *coli* resistance to H_2_O_s_ stress [65]. *IbeA* is a necessary component for invasion of IECs and absence or mutation of this gene limits survival of AIEC within macrophage[66]. The type VI secretion system has been implicated in targeting other bacterial and eukaryotic cells [67]. We found homologous CDS for *chuA* and *yersiniabactin*, in 6 of 11 NIEC. These iron uptake genes are enriched among AIEC strains [37] and other pathogenic E. coli including ExPEC and EHEC. However, it remains to be determined whether these genes are expressed in the noninvasive strains. This analysis is limited by the fact that growth in pure cultures represents a very different environment than within the human intestine, and thus does not take into consideration complex microbe-microbe and host-microbe interactions. In addition, our subtractive genomics approach was limited to CDS expressed in the reference AIEC strain LF82. NRG857C has a genome that is highly similar to LF82 however, CDS in NRG857C but absent in LF82 were present in as many as six of the 13 other AIEC strains. Additional CDS that were homologous among three or more AIEC except LF82 and absent in the 11 NIEC are listed in S3 Table. Nonetheless, the results of this analysis provide a useful baseline repertoire of *E*. *coli* transcriptional patterns that may aid in the analysis of complex patient based metatranscriptomic data.

Among the 166 CDS mapping to the LF82 genome, we identified four potential signature transcripts belonging to CRISPR-associated (Cas) genes. These genes map to a region of the LF82 genome that is highly specific [37] and in our analysis these CDS were conserved in 4 of 6 AIEC strains. We did not find homologous CDS in DEC, although they are homologous to CDS in three ExPEC. Among the strains with these specific Cas genes, four of the AIEC strains and the three ExPEC are of the B2 phylotype. AIEC of the B2 phylotype are described to be among the most abundant and the most virulent [68]. CRISPR-Cas forms the adaptive immunity system [69–71]. Bacterial strains express Cas proteins that recognize foreign genetic elements in plasmids and phages and insert fragments of the exogenous DNA into their own genomes. Most *E*. *coli* harbor CRISPR-Cas systems that belong to subtype I-E [72]. LF82 has the I-F system which has 3 CRISPR arrays and an operon of 6 cas-F genes (*cas6f*, *csy3*, *csy2*, *csy1*, *cas2*, *cas3*, and *cas1*) [72]. This system is also found in *Yersinia pestis* an enterotoxigenic *E*. *coli* (strain B7A) and a subset of B2 phylotype *E*. *coli* [72]. Toro et al, [73] examined the relationship between CRISPR-Cas systems and virulence in Shiga toxin-producing *E*. *coli* (STEC) and observed conservation of CRIPR spacer contents among strains of the same serotype and that the highly virulent STEC strains had fewer spacers within CRISPR arrays. Two other groups have recently identified CRISPR-Cas gene loci for the development of serotype-specific PCR assays of STEC [74, 75] and *Salmonella enterica* serotypes Typhi and Paratyphi A [76].

We analyzed 53 fecal samples (Table 6) using 4 Cas gene assays and 35.7% of CD compared to 6.2% of non-IBD control samples (p = 0.02) revealed positive assays for 3 of the 4 assays. Using the *pduC* primers described in Dogan et al[34], expression of the *pduC* gene was detected in 21.4% CD compared to 25% of non-IBD controls (p = 1.0). For the excisionase gene 64% of CD compared to 44% of non-IBD control samples (p = 0.34) had positive assays. We also analyzed 38 parallel ileal biopsy samples (Table 7) and 50% of CD compared to 13% of non-IBD control samples (p = 0.04) had positive assays. Expression of the *pduC* gene was detected in 17% CD compared to 4% of non-IBD controls (p = 0.27). For the excisionase gene 33% of CD compared to 0% of non-IBD control samples (p = 0.0095) had positive assays. All 4 excisionase positive samples were correspondingly positive for Cas genes. The p-values for the Cas assays did not reach significance after applying the Bonferroni correction for multiple comparisons (p <0.01). Nevertheless we observed a similar trend in fecal and/or ileal biopsies for all four of the Cas genes tested. Our data suggests the Cas genes may serve as promising AIEC biomarkers; this will need to be confirmed in a larger set of patient samples. We did not detect a significant difference in *E*. *coli* 16S rRNA gene expression (ΔCT) relative to total bacteria in cases compared to non-IBD controls.

Altogether our sample sizes were small and *pduC* expression was less discriminating for AIEC infected samples. However, it may be a useful target for therapeutic intervention as previously described. It is also possible that other *pdu* operon genes are more specific and could serve as better targets. Our study is consistent with other reports that no single gene is able to distinguish AIEC from NIEC. Furthermore, it remains to be demonstrated whether any candidate AIEC signature transcripts with utility as a microbial biomarker, has a functional role in pathogenicity.

In summary, these results identify potential candidate AIEC signature transcripts, which may be more prevalent among CD patients than non-IBD patients and serve as proof of principle for our comparative genomic/transcriptomic analysis of AIEC and NIEC.

## Supporting Information

S1 Fig Specific CAS genes are detected in AIEC by PCR. Agarose gel electrophoresis analysis of PCR products obtained from reactions using forward and reverse primers of the Cas genes LF82_091 and LF82_092, with E. coli 16S rRNA as a positive control. Positions of molecular size standards (in bp) are indicated, also see methods.
(TIF)

S2 Fig Specific CAS genes are detected in AIEC by PCR. Agarose gel electrophoresis analysis of PCR products obtained from reactions using forward and reverse primers of the Cas genes LF82_088 and LF82_093, with E. coli 16S as a positive control. Positions of molecular size standards (in bp) are indicated, also see methods.
(TIF)

S1 File Up-regulated and Down regulated transcripts in LF82 and HS. **Table A. Up-regulated transcripts in LF82 compared to HS.** RNA was extracted from bacteria at exponential (2h) and stationary (24h) phases of growth in pure cultures and RNA sequencing completed. Expression level of homologous CDS in LF82 and HS is compared at 2 h, 24h and at both time points using edgeR. Up-regulated CDS with fold change ≥ 2, FDR <0.05. The RPKM values for LF82 and HS are shown. **Table B. Down-regulated transcripts in LF82 compared to HS cultures.** RNA was extracted from bacteria at exponential (2h) and stationary (24h) phases of growth in pure cultures and RNA sequencing completed. Expression level of homologous CDS in LF82 and HS is compared at 2 h, 24h and at both time points using edgeR. Up-regulated CDS with fold change ≥ 2, FDR <0.05. The RPKM values for LF82 and HS are shown.
(XLSX)

S2 File GO functional categories in LF82 and HS. Table A. Up-regulated GO-categories (FDR <0.05) in LF82 compared to HS cultures at 2h. Table B. Up-regulated GO-categories (FDR < 0.05) in LF82 compared to HS cultures at 24h. Table C. Down-regulated GO categories (FDR <0.05) in LF82 compared to HS cultures at 2h. Table D. Down-regulated GO categories (FDR <0.05) in LF82 compared to HS cultures at 2h.
(XLSX)

S1 Table LF82 transcripts that lack homology (< 85% sequence identity) within 11 non-invasive *E*. *coli* strains. (XLSX)

S2 Table Forward and reverse primers for RT-qPCR assays. (DOCX)

S3 Table AIEC genes that do not share sequence homology (<85% sequence identity) with LF82 or non-invasive *E*. *coli* strains. (XLSX)
