# Correction: Identification of Candidate Adherent-Invasive *E*. *coli* Signature Transcripts by Genomic/Transcriptomic Analysis

**DOI:** 10.1371/journal.pone.0134759

**Published:** 2015-07-28

**Authors:** Yuanhao Zhang, Leahana Rowehl, Julia M. Krumsiek, Erika P. Orner, Nurmohammad Shaikh, Phillip I. Tarr, Erica Sodergren, George M. Weinstock, Edgar C. Boedeker, Xuejian Xiong, John Parkinson, Daniel N. Frank, Ellen Li, Grace Gathungu

The following information is missing from the Funding section: Dr. Tarr is also supported by the National Institutes of Health [5P30 DK052574 (DDRCC)]. The funders had no role in study design, data collection and analysis, decision to publish, or preparation of the manuscript.
