# Supplementary figures and images for: Identification of Candidate Adherent-Invasive E. coli Signature Transcripts by Genomic/Transcriptomic Analysis
Source: PLoS One. 2015 Jun 30;10(6):e0130902. doi: 10.1371/journal.pone.0130902 (PMC4509574; doi:10.1371/journal.pone.0130902)

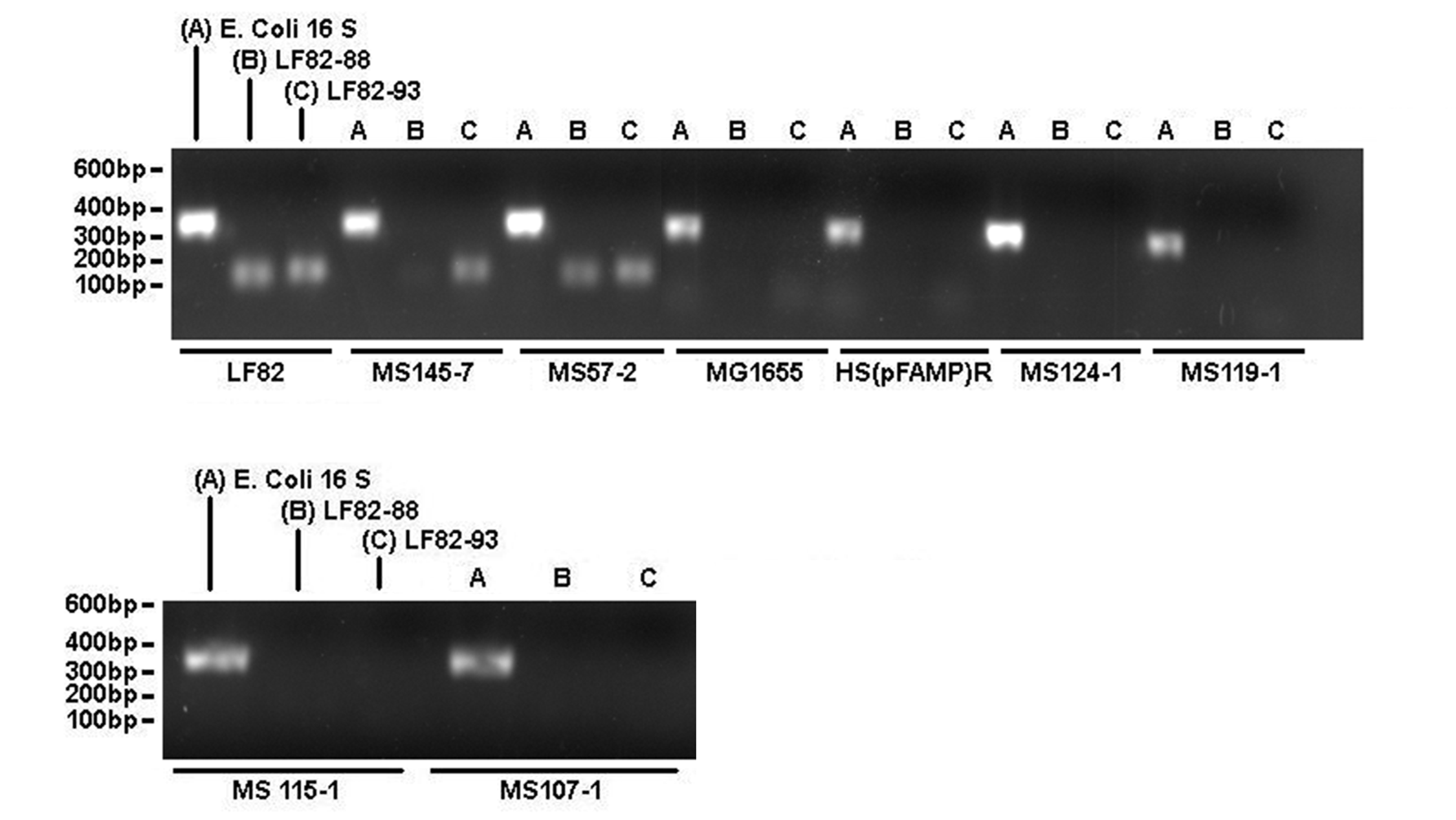

Supplement: S1 Fig — Agarose gel electrophoresis analysis of PCR products obtained from reactions using forward and reverse primers of the Cas genes LF82_091 and LF82_092, with E. coli 16S rRNA as a positive control. Positions of molecular size standards (in bp) are indicated, also see methods. (TIF) [file pone.0130902.s001.TIF]

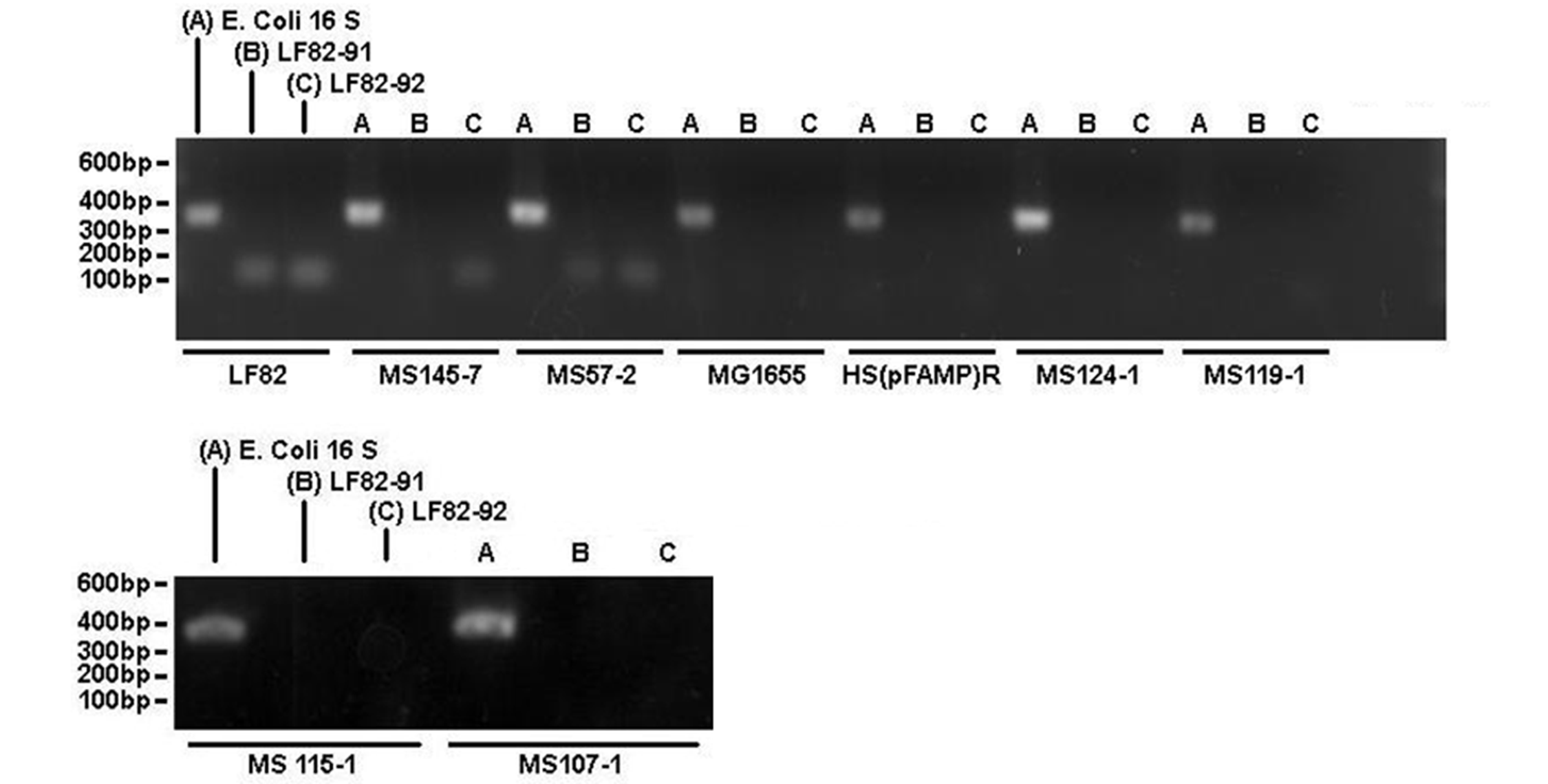

Supplement: S2 Fig — Agarose gel electrophoresis analysis of PCR products obtained from reactions using forward and reverse primers of the Cas genes LF82_088 and LF82_093, with E. coli 16S as a positive control. Positions of molecular size standards (in bp) are indicated, also see methods. (TIF) [file pone.0130902.s002.TIF]
