# Supplementary material for: Identification of Candidate Adherent-Invasive E. coli Signature Transcripts by Genomic/Transcriptomic Analysis
Source: PLoS One. 2015 Jun 30;10(6):e0130902. doi: 10.1371/journal.pone.0130902 (PMC4509574; doi:10.1371/journal.pone.0130902)
Supplement: S2 Table — (DOCX) [file pone.0130902.s006.docx]

**S2 Table. Forward and reverse primers used for PCR analysis.**

| Genes | Primer sequence (5′à 3′) | Expected amplicon size (bp) | Reference |
| --- | --- | --- | --- |
| LF82_088 | TGGTCCAGCTCGAAAATGCT  TGGTTTTCTGCCGCGTCTAT | 107 | This study |
| LF82_091 | GGCAGCATTGTCAGTATGCG  GCTCGTAAAGCGCATAACCC | 109 | This study |
| LF82_092 | GCCGAACAGATCCAGGTGAA  CTGCCTGTCCCTGGTCAAAT | 97 | This study |
| LF82_093 | GGCGTAAAGGGCTGACAGAT  TGAACGCCTTAACAGACGCT | 125 | This study |
| LF82_095 | GCCCAAGAAGCCTTGAAACA  GCTGATTCGTGGAACAGATA | 100 | This study |
| *pduC* | CCTGCAAAAATCGTCGAAGTGGTT  TTGGTGACGTGAGCCTGTTGTGAT |  | Dogan et al,[34] |
